# Low-Income Students in Higher Education: Undermatching Predicts Decreased Satisfaction toward the Final Stage in College

**DOI:** 10.1007/s10964-019-01022-1

**Published:** 2019-04-19

**Authors:** Marjolein Muskens, Willem E. Frankenhuis, Lex Borghans

**Affiliations:** 1 School of Business and Economics, Maastricht University, Tongersestraat 53, 6211 LM Maastricht, The Netherlands; 2 Behavioural Science Institute, Radboud University, Montessorilaan 3, PO Box 9104, 6500 HE Nijmegen, The Netherlands

**Keywords:** Socioeconomic status, Students, Undermatching, Social mobility, Satisfaction, Propensity score matching

## Abstract

It is undesirable when students attend institutions that are less selective than their academic credentials would permit (i.e., undermatching) because of the long-term consequences for their job opportunities and wages, in particular for students from low-socioeconomic (SES) backgrounds. Undermatching may also affect students’ satisfaction during college. Research from a life course perspective shows that subjective experiences during college may have long-term impact on adolescents’ development. However, little is known about the relation between undermatching and students’ subjective experiences during their years in college, and about whether this relation is moderated by SES. From an academic misalignment perspective, undermatching may lead to less satisfaction because undermatched students are not maximizing their potential. However, from a social misalignment perspective, experiences of social mismatch when low-SES students enter the most selective institutions are well documented, and such mismatch may be less pronounced in less selective institutions. Consequently, there may be a positive relation between undermatching and satisfaction with the social environment for low-SES students. The current study tested these relations by using propensity score matching (PSM) to analyse the association between undermatching, SES, and satisfaction among 21,452 respondents (67% female) among 1^st^, 2^nd^, 3^th^, and 4^th^ year college students from a cohort study among students in the Netherlands (Dutch Student Monitor), all of whom were eligible for the most selective institutions. The results indicated a negative relation between undermatching and satisfaction with the social and academic environment, both for low- and high-SES students. The negative relation between undermatching and both forms of satisfaction increases toward the last year in college, especially for low-SES students. This lowered satisfaction in the final stage in higher education implies that the negative consequences of undermatching become more pronounced after students have become more integrated in their colleges. These findings have important implications for the understanding about undermatching in relation to students’ development and for the formulation of policies and programs for promoting social mobility.

## Introduction

Graduating from the most selective institutions is related to more career opportunities and higher wages in the long term compared to graduating from less selective institutions (Mayhew et al. 2016). Because of these economic benefits of college completion in selective institutions, undermatching, when students attend institutions that are less selective than their academic achievements would enable, is an undesirable outcome (Tiboris 2014). In addition, from an academic misalignment perspective, undermatching leads to less positive college experiences than matching. Because of the less rigorous study program in less selective institutions, high-achieving undermatched students would be less challenged to develop their full potential (Hoxby and Turner 2013). This mismatch may lead to less academic integration, contributing to the risk of dropping out of college (Bowen et al. 2009). The recurrent finding that undermatching is negatively related to college completion (Ovink et al. 2018) supports this hypothesis. Importantly, not all groups are equally likely to undermatch. Social scientists and policymakers are particularly worried about the high prevalence of undermatching among students from low-socioeconomic (low-SES) backgrounds (Howell and Pender 2016), because this leads to reinforcement of social and economic inequality (Deutschlander 2017).

Research from a life course perspective has shown that early circumstances and experiences can have cumulative and enduring consequences for life experiences in later developmental stages (Yoshioka and Noguchi 2009). More specifically, adolescents’ subjective experiences during their years in college may have far-reaching consequences for their development toward adulthood, for example for academic achievement and psychosocial well-being (Wickrama et al. 2016). However, little is known about the subjective experiences of students who undermatch. The present study will investigate the relationship between undermatching, socioeconomic status, and students’ subjective experiences.

### Socioeconomic Status

An extensive body of research has shown that students’ SES is one of the strongest correlates of academic performance (e.g., Sirin 2005). In addition, the size of the relationship between SES and educational attainment increases by each school level, suggesting that the gap between low- and high-SES students in academic achievement expands through students’ lives (Sirin 2005). The relation between SES and educational achievement is related to several factors, such as differences in cognitive development related to harmful effects of poverty (Mullainathan and Sharif 2013), low-SES students’ higher chance to encounter lower educational expectations from their parents (Davis-Kean 2005), and their teachers (Jussim et al. 1996), and a higher likelihood of being placed in low-resource schools compared to high-SES students (Lee and Burkam 2002). Moreover, researchers have also suggested that there may be a mismatch between the social-cognitive skills that children from disadvantaged circumstances develop in interaction with their home environments, and social-cognitive skills demanded in school-environments (Ellis et al. 2017). For example, in higher education, a cultural mismatch with regard to norms in interacting with others seems to fuel social-class disparities (Stephens et al. 2018).

### Student experiences

Theoretical models of student experiences in college distinguish between integration in the social and academic system. In the classical work of Tinto (1975), integration in these two systems was defined as primary condition for student success. The social environment refers to social aspects of college, such as interactions with fellow students and teachers, whereas the academic environment refers to intellectual aspects of college, such as the content and structure of the study program. Indeed, decades of research testing Tinto’s model in different college systems and environments, has shown that when examining students’ integration, it is valuable to distinguish between the social and academic environment (Aljohani 2016).

Notably, theories about cultural capital predict difficulties with integration in the social environment when low-SES youth enter the most selective institutions, and the existence of these social barriers is well documented (Jury et al. 2017). Differences in cultural capital and codes between low- and high-SES students (Jæger 2009), signalled by teachers and fellow-students, might lead to social exclusion of low-SES students (Walpole 2003). This experience of not fitting in can lead to lower levels of satisfaction with the social environment in the most selective tracks (Jury et al. 2017). This mismatch may be less present in less selective institutions because of the presence of more low-SES students (Bastedo and Jaquette 2011), and, as a consequence, cultural codes that may match better with low-SES students’ backgrounds (Walpole 2003). At the same time, it is also suggested that undermatching leads to a misalignment between students’ academic capacities and demands from the educational program. This misalignment may lead to less satisfaction with academic aspects in college (Hoxby and Turner 2013), and difficulties with integration in the academic environment (Aljohani 2016). By contrast, in the most selective tracks, highly qualified students may be more likely to experience an academic match (Hoxby and Avery 2013).

Student integration is a developmental process, and classical research on students’ integration suggests that students go through several stages after their transitions into college. First, there is the stage of separation when students start to detach themselves from the norms and values of their previous communities, such as family and high school. Second, students go through the phase of transition, when they adapt new norms and value from their college. Third, in the phase of incorporation, students are successfully integrating in the new community (Aljohani 2016). Therefore, in studying student experiences, it is important to incorporate a time perspective.

### Undermatching in the Netherlands

Research on the phenomenon of undermatching has been conducted mainly in the educational context of the U.S. To determine undermatching, researchers have to define institutions’ selectivity levels and, in addition, they have to determine which students are eligible to gain admission to these levels. These distinctions are in practice gradual. Because researchers have used different approaches in defining both elements, estimating undermatching has leaded to different results, such as a wide range of undermatch rates, and conflicting findings for underrepresented students (Rodriguez 2015). By focussing on undermatching in the Netherlands, these issues are circumvented, since the Dutch education system has a clear distinction between two levels of higher education, with almost no variation in quality within each level, and there are clear rules who can attend the higher level. On other aspects, the Dutch situation is very similar to higher education in the U.S. and in Europe. Both in the U.S. and in Europe, high school (or secondary education) is followed by postsecondary (or tertiary) education, generally during late adolescence. The contexts are similar regarding academic degrees that can be attained, and educational and professional careers during and after higher education. In addition, patterns of educational inequality, such as the tendency among low-SES students to undermatch (Dutch Inspectorate of Education 2018), are comparable between the U.S and the European context.

The main differences the current study takes advantage of are differences in admission procedures and institutions selectivity. First, there are differences with regard to admission procedures to higher education. In the U.S, institutions for higher education determine their own admission standards, and therefore, it depends on the specific higher education institution who is admitted and who is not. Students’ eligibility is determined in the last stage of high school, with performance on standardized tests (i.e., SAT, ACT), and by assessing their academic and extracurricular performance. In the Netherlands, as in many other European countries, students’ eligibility for higher education is determined by students’ track in secondary education, and all institutions of higher education follow these standards. In the Netherlands, the eligibility for the most selective institutions is determined by having completed the highest secondary track (i.e., VWO), whereas participating in a lower track (i.e., HAVO) leads to eligibility to less selective institutions. Therefore, in the Netherlands, undermatching is a consequence of students’ choice to attend a less selective institution after completing the highest secondary track. The prevalence of undermatching among students with the eligibility to attend the most selective institutions ranges between 10 and 13% (Van den Broek et al. 2018).

Second, there is an important difference between the U.S. context and the European context regarding higher education institutions’ selectivity. Whereas in the U.S. context, selectivity is often seen as a continuum (Roderick et al. 2006), in the Dutch context there are only two types of higher education institutions: most selective institutions and less selective institutions. In the U.S. context, both students’ admissibility and institutions’ selectivity have to be empirically estimated, leading to discussion about the accuracy of the estimation of undermatching (Smith et al. 2013). By contrast, in the Dutch context, both students’ admissibility and institutions’ selectivity are fully transparent and clearly defined, and therefore undermatching is easy to determine. Respondents are determined as ‘matched’ when they are eligible for the most selective institutions and subsequently enrolled in an institution that can be characterized as ‘most selective’. Respondents are determined as ‘undermatched’ when they are eligible for the most selective institutions and subsequently have chosen to enrol in an institution that is less selective.

## Current Study

The predominant view is that undermatching is undesirable and has negative long-term consequences for wages and job status. Subjective experiences during college are also important because of their relation with college completion and, from a life course development perspective, because these experiences accumulate and may have enduring consequences during adulthood. However, little is known about the relation between undermatching and subjective experiences of students during their years in higher education, and about the possibility that students’ SES plays a moderating role in this relation. Using a representative sample of 22,521 adolescents, the current study attempts to enhance knowledge about these relations and their development over the course of students’ time in higher education. From an academic misalignment perspective, undermatching leads to less satisfaction because of the mismatch between students’ high capacity and the less rigorous educational program in less selective institutions. However, from a social misalignment perspective, undermatching may have differential effects for low- and high-SES students, predicting more satisfaction for students with a low-SES background because of less experiences of social mismatch in the less selective institutions than in the highly selective institutions. In addition, the role of students’ time spent in college (i.e., 1^st^, 2^nd^, 3^th^ and 4^th^ year) in these relationships is explored.

## Methods

### Participants and Data Set

Research questions, inclusion criteria, sample size, and constructs of this study are preregistered (Frankenhuis & Nettle, 2018) at Open Science Framework (https://osf.io/4x5p8/). During the review procedure, this study has deviated from the preregistration on several aspects, reported in an addendum added to the preregistration (https://osf.io/crhjx/). This section reports how the sample size was determined, all data exclusions, and all measures in the study.

Data for this study come from the Dutch Student Monitor, a large-scale survey of youth’s experiences during their time in higher education in the Netherlands. Participants were selected with a random sample procedure from all higher education institutions in the Netherlands. The dataset used for this study contains data from 3 waves (from 2013 to 2015; *N* = 58,177). To minimize pre-existing differences in skills and cognitive abilities, only respondents were included who are eligible for the most selective institutions (i.e., about 45% of this sample), who are enrolled in higher education (i.e., university or higher professional education) in a full-time educational program, and who are between 17 and 25 years old (*M* = 21.58, *SD* = 1.77). Therefore, the final sample includes 21,452 students who are all eligible for the most selective institutions. The majority (87%) chose to enrol in the most selective institutions (i.e., matched students), and a minority (13%) chose to enrol in less selective institutions (i.e., undermatched students).

### Measures

#### Satisfaction

The outcome measure of interest is student satisfaction. Because of the theoretical model for student integration that this study builds on (Davidson and Wilson 2013), two scales for satisfaction were applied: (1) satisfaction with the social environment, and (2) satisfaction with the academic environment. In the Student Monitor survey, participants responded to 20 single items on satisfaction. From these items, drawing on this theoretical model, three items were selected, that reflect their experiences with the social environment, and three items were selected that capture their experiences with the academic environment. Students responded using a 5-point Likert scale (1 = very dissatisfied, 5 = very satisfied) assessing how satisfied they are with several aspects regarding their experiences in college. The scale for social satisfaction was constructed from the items regarding satisfaction with: (1) the general atmosphere, (2) students’ attitude toward fellow students, and (3) teachers’ attitude toward students. The scale for academic satisfaction was constructed from the items regarding satisfaction with: (1) the content of the programme, (2) general skills learned in the programme, and (3) the degree to which the educational program is academically challenging. The two scales were reidentified by a joint factor analysis, explaining 65% of the variance. Internal consistencies (Cronbach’s Alpha) were .72 for both scales.

#### Match and Undermatch

The treatment variable is undermatching. In the Netherlands, students are either eligible for the most selective institutions of higher education (i.e., they attained a diploma in the highest level in high school) or not (i.e., they have not attained a diploma in the highest level in high school^1^). In addition, there are two types of institutions in higher education: most selective institutions (i.e., students are only admitted when they have attained a diploma in the highest level of high school^2^), and less selective institutions (i.e., a diploma from the highest level of secondary education (i.e., VWO) is not required, a diploma in a lower level of secondary education (i.e., HAVO) gives eligibility). Respondents were determined as ‘matched’ when they are eligible for the most selective institutions in higher education and subsequently choose to attend an institution that can be characterized as ‘most selective’. Respondents are determined as ‘undermatched’ when they are eligible for the most selective institutions in higher education and subsequently have chosen to attend an institution that is less selective.

#### Socioeconomic Status

SES was measured in two ways. Because various components of SES could work through different processes when affecting health and psychosocial outcomes, using more than one single measure of SES is important. Following recommendations by the APA task force Socioeconomic Status (2007), an objective and a subjective measure of SES was employed. Research has shown that subjective social status is correlated with well-established measures of SES, but also that it may capture unique aspects socioeconomic circumstances that predict outcomes related to well-being often missed by objective indicators of SES (Bradshaw et al. 2017). This study thus included: (1) an objective measure based on parental educational level with two levels: (1, Low-SES), both parents have not obtained a diploma in higher education, (0, High-SES), one or both parents obtained a diploma in higher education, and (2) a subjective measure (Singh-Manoux et al. 2003) based on a single item regarding students’ view of their parents’ social class (“Some people belong to a higher social class, others to a lower one. Considering your own social background, where on the scale would you place your parents/caregivers?”). The response scale ranged from 1, lower social class, to 10, higher social class.

### Covariates

#### Grades

In Dutch education, the grading system consists of marks from 1 (very poor) to 10 (outstanding). The pass mark for a single course is ‘6’. Students’ average grades were measured by asking them to report their average grade during their last year in high school using a single-item question. Self-reported grades are reasonably good reflections of actual grades, especially among high-performers, and are strong predictors of future grade points (Kuncel et al. 2005).

#### Motivation Before College

To control for the potential confounding effects of differences in students’ motivation before their transition to higher education, respondents were asked how motivated they were before going to college to attain a degree in higher education on a 5-point Likert scale (1 = not at all motivated, 5 = very motivated).

#### Additional Controls

Other covariates in the models include indicators for gender, age, immigrant status (i.e., at least one parent was born abroad) (with non-immigrant status as reference category), the language spoken at home with parents (with Dutch as reference category), students’ disability (measured with a question about suffering from a disability or chronicle disease (yes = 1, no = 0), and college major. Descriptive statistics for all variables are displayed in Table 1.

**Table 1:** Descriptive statistics among study variables

	1	2	3	4	5	6	7	8	9	10	11
1 Satisfaction (social)											
2 Satisfaction (academic) environment	0.47*										
3 Undermatched	−0.07*	−0.10*									
4 SES def. 1	−0.03*	−0.03*	0.08*								
5 SES def. 2	0.05*	0.06*	−0.04*	−0.41*							
6 Male	0.02*	0.01	−0.05*	0.01	−0.03*						
7 Age	−0.05*	−0.04*	0.00	0.02*	0.01	0.07*					
8 Immigrant status	−0.07*	−0.02*	−0.03*	0.05*	−0.11*	0.02*	0.03*				
9 Home language Dutch	0.06*	0.02*	0.01*	−0.05*	0.10*	−0.01	−0.03*	−0.35*			
10 No disability/disease	0.03*	0.02*	−0.02*	−0.01	0.05*	0.07*	−0.03*	0.00	0.00		
11 Grade in high school secondary education	0.08*	0.08*	−0.22*	−0.12*	0.08*	0.02*	0.00	−0.02*	−0.03*	0.05*	
12 Motivation before college	0.16*	0.17*	0.04*	0.00	0.03*	−0.09*	−0.03*	−0.01	0.00	−0.01	0.09*
13 Years in higher education	−0.01	−0.04*	−0.07*	0.01	0.01*	0.03*	0.78*	−0.02*	0.00	0.03*	0.12*
14 Economics	−0.06*	−0.05*	0.12*	0.03*	0.01	0.08*	−0.05	0.03*	−0.04*	0.04*	−0.02*
15 Education	0.01	−0.02*	0.27*	0.01	0.00	−0.05*	0.00*	−0.02*	0.01	−0.01*	−0.06*
16 Agriculture	0.11*	0.04*	−0.05*	−0.01*	0.00	−0.03*	−0.02*	−0.04*	0.01*	−0.02*	−0.04*
17 Nature	0.06*	0.04*	−0.12*	−0.03*	0.00	0.08*	0.00	0.02*	0.00	−0.01*	0.09*
18 Science	0.01	0.03*	0.02*	−0.01	0.00	0.28*	0.01	−0.02*	0.02*	0.01*	0.04*
19 Health	−0.05*	0.04*	0.01*	−0.02*	0.05*	−0.15*	0.06*	0.02*	0.00	0.02*	0.07*
20 Law	−0.06*	−0.02*	−0.07*	0.03*	0.00*	−0.02*	−0.01	0.03*	−0.03*	−0.01*	−0.03*
21 Behavior	−0.04*	−0.08*	−0.03*	0.02*	−0.03*	−0.14*	−0.01	0.00	0.02*	−0.01*	−0.14*
22 Language	0.05*	0.01	−0.03*	0.00	−0.03	−0.07*	0.01	−0.03*	0.00	−0.04*	0.03*
Mean or Proportion	4.10	3.85	0.13	0.31	6.78	0.33	21.58	0.06	0.92	0.72	7.21
SD	0.61	0.64	0.34	0.46	1.50	0.47	1.77	0.23	0.27	0.45	0.64
Minimum	1	1	0	0	1	0	17	−1	0	0	5.51
Maximum	5	5	1	1	10	1	25	1	1	1	10
	12	13	14	15	16	17	18	19	20	21	22
1 Satisfaction (social)											
2 Satisfaction (academic) environment											
3 Undermatched											
4 SES def. 1											
5 SES def. 2											
6 Male											
7 Age											
8 Immigrant status											
9 Home language Dutch											
10 No disability/disease											
11 Grade in high school secondary education											
12 Motivation before college											
13 Years in higher education	−0.03*										
14 Economics	−0.04*	−0.04*									
15 Education	0.03*	−0.01	0.00								
16 Agriculture	−0.01	−0.01	0.00	0.00							
17 Nature	−0.02*	0.01	0.00	0.00	0.00						
18 Science	−0.03*	0.01	0.00	0.00	0.00	0.00					
19 Health	0.07*	0.06*	0.00	0.00	0.00	0.00	0.00				
20 Law	−0.03*	−0.02*	0.00	0.00	0.00	0.00	0.00	0.00			
21 Behavior	−0.01	−0.01	0.00	0.00	0.00	0.00	0.00	0.00	0.00		
22 Language	0.04*	−0.02*	0.00	0.00	0.00	0.00	0.00	0.00	0.00	0.00	
Mean or Proportion	4.29	2.61	0.14	0.02	0.06	0.11	0.15	0.19	0.05	0.16	0.11
SD	0.78	1.16	0.34	0.13	0.24	0.32	0.36	0.40	0.22	0.37	0.32
Minimum	1	1	0	0	0	0	0	0	0	0	0
Maximum	5	4	1	1	1	1	1	1	1	1	1

*N* = 21,452

*p < 0.05

### Analytic Plan

In testing the relation between undermatching in student satisfaction, selection effects might play a role. Students who choose to undermatch may differ from students who choose to go the most selective institutions on several characteristics. For example, it is possible that undermatched students, compared to matched students, are likely to be less motivated to attain a degree before their entrance to college. In this way, matched and undermatched students differ on salient characteristics that spuriously inflate the effect that undermatching would have on the development of satisfaction when using traditional methodological techniques. For that reason, propensity score matching (PMS; Thoemmes and Kim (2011)) was applied. PSM creates a treatment group (in the current study, students who are undermatched) and a comparison group (in the current study, students who are matched), and compares these groups on the outcome measure of interest (in the current study, student satisfaction). PSM creates two equal groups by matching them on several covariates that may affect the propensity to undermatch and student satisfaction (Rosenbaum and Rubin 1983). More specifically, PSM estimates a logit model predicting the treatment (i.e., undermatching) with covariates. In this study, the propensity to undermatch was first estimated with a logit model (conducted with R essentials for SPSS 24) that predicts undermatch with the covariates described earlier (except for the covariate college major, because experiences with college major occurred after being matched or undermatched). Each respondent was assigned a propensity to undermatch, varying from propensity scores close to 0 (very low chance to undermatch) to 1 (very high chance to undermatch). Second, respondents from the treatment group (i.e., undermatched students) were matched with respondents from the comparison group (i.e., matched students) using the propensity scores. In the analyses presented, the two-to-one nearest-neighbour matching technique was applied, without replacement and with a conservative 0.02 caliper level. The two-to-one nearest-neighbour matching technique was the most suitable approach because there are more untreated (matched) respondents (*N* = 18,626) than treated (undermatched) respondents (*N* = 2,826) in the dataset, and the 2:1 ratio is found to improve precision without a commensurate increase in bias (Austin 2010). (However, estimating models with the specification one-to-one nearest neighbour matching appeared to show the same results as the two-to-one nearest neighbour matching, results not shown). Each undermatched student was matched to a maximum of two matched student based on their propensity score, and the difference between these two respondents was not larger than 0.02. This resulted in 2826 students in the treatment and 5507 respondents in the comparison group.

After following the PSM procedure, the relation between undermatching, SES, and satisfaction was tested with linear regression models, applying satisfaction (with the social and academic environment, separate analyses) as dependent variable, undermatching, SES, and the interaction undermatching × SES as predictors, and college major as covariate (eight dummy variables). In addition, in order to assess the development of these relationships during the years in college, the predictor year, and the interaction undermatching × SES × year (and all relevant two-way interactions) were added to the model. Finally, the simple mean differences with regard to year, SES and undermatching were evaluated. The presentation of the results is focuses on results of interactions in linear regression models (conducted with software package SPSS 24), as these assess the research questions.

## Results

Descriptive statistics and correlations among study variables are shown in Table 1.

The analysis began by estimating a logit model by predicting undermatching with nine covariates. Most of the covariates were statistically significant predictors of undermatching (see Table 2).

**Table 2:** Logistic regression estimates for propensity score models

Variable	Coefficient (Exp B)	Standard error	*p*-Value
SES def. 1 (1 = first generation)	1.42**	0.05	<0.000
SES def. 2 (subjective social class)	0.99	0.02	0.565
Gender (1 = male)	0.74**	0.05	<0.000
Age	1.02	0.01	0.156
Immigrant status (1 = immigrant status)	0.54**	0.11	<0.000
Home language (1 = Dutch)	0.96	0.09	0.668
Disability or chronicle disease (1 = no)	0.99	0.05	0.775
Average grade high school	0.19**	0.62	<0.000
Motivation before entrance to higher education	1.24**	0.03	<0.000
Constant	919.70**	2.20	<0.000

*N* = 21,452

*p < 0.05, **p < 0.01

Overall, five of the covariates reached statistical significance (*p* < 0.01, two-tailed tests). Covariates that were not a significant predictor of undermatching, such as age, were still included in the final model to create the propensity score.

Next, a two-to-one nearest-neighbour matching technique was conducted without replacement and with a 0.02 caliper level (i.e., matched neighbours differ no more than 0.02 standard deviations of the logit of the estimated propensity score). Results indicate that the matching was successful, with all covariates having a standardized mean difference smaller than 0.25 after matching. In addition, the Relative Multivariate Imbalance test showed a lower L1 after matching (.670) than before matching (.732) (Iacus et al. 2011), indicating that the matching procedure generated balance.

Table 3 shows the results of the pre- and postmatching t-tests, examining differences between undermatched and matched students on the covariates. In the left-hand columns (i.e., the unmatched sample), can be seen that matched and undermatched students differ significantly on six out of nine covariates. These significant differences show that the results may be confounded when analysing the relationship between undermatching and students’ satisfaction, and that PSM may address this issue. In the right-hand columns (i.e., the matched sample), the results are shown of t-tests after matching on the propensity score. The results show that matching was indeed successful, because it eliminated all significant differences on covariates between matched and undermatched students.

**Table 3:** Achieving balance among undermatched and matched students: Pre- and Post-test matching T-tests using 2-1 nearest neighbour matching

	Unmatched sample			Matched sample		
	Control (Matched)	Treatment (Undermatched)	*t-*value	Control (Matched)	Treatment (Undermatched)	*t-*value
SES def. 2 (1 = first generation)	0.29	0.41	12.08**	0.39	0.41	1.62
SES def. 2 (subjective social class)	6.80	6.65	−5.27**	6.62	6.65	0.69
Gender (1 = male)	0.34	0.27	−7.48**	0.28	0.27	−1.01
Age	21.58	21.59	0.10	21.58	21.59	0.19
Immigrant status (1 = immigrant status)	0.06	0.04	−4.53**	0.04	0.04	−0.70
Home language (1 = Dutch)	0.92	0.93	1.73	0.92	0.93	0.68
Disability or chronicle disease (1 = no)	0.73	0.70	−2.56*	0.71	0.70	−0.51
Average grade high school	7.26	6.84	−33.55**	6.87	6.85	−1.94
Motivation before entrance to higher education	4.28	4.37	5.76**	4.36	4.37	0.54
*N*	18626	2826		5507	2826	

*p < 0.05, **p < 0.01

Because the PSM procedure generated balance between undermatched and matched students with regard to covariates, next, the matched dataset was appropriate for examining the interaction between undermatching and SES in relation to student satisfaction. The results of the final analyses, with satisfaction as outcome measure, undermatching, SES, and the interaction undermatching × SES as predictor, and college major as covariates, are shown in Table 4. The results show no interaction between undermatching and SES. Moreover, undermatching is related to less satisfaction with the academic and social environment. These relations are significant with modest effect sizes. These results indicate that regardless of SES, undermatching is related to lower satisfaction during college. These results show no evidence suggesting that undermatching may take along benefits in terms of more satisfaction for students with a low-SES background.

**Table 4:** Estimated coefficients (Beta) predicting student satisfaction after propensity score matching

	Outcome: Satisfaction with the social environment		Outcome: Satisfaction with the academic environment	
	β	SE	β	SE
Undermatching (1 = undermatched)	−0.12**	0.02	−0.18**	0.02
Low-SES (1 = low)	−0.01	0.02	−0.03	0.02
Interaction: Low-SES × Undermatching	0.01	0.03	0.03	0.03
Constant	3.98**	0.02	3.78**	0.02
R-sq	0.03		0.03	
*N*	8333		8333	

Standardized coefficients. Both models control for college major (8 dummies, results not shown)

*SES* socioeconomic status

*p < 0.05, **p < 0.01

### Undermatching, SES, and student satisfaction during four years in higher education

**Fig. 1:**
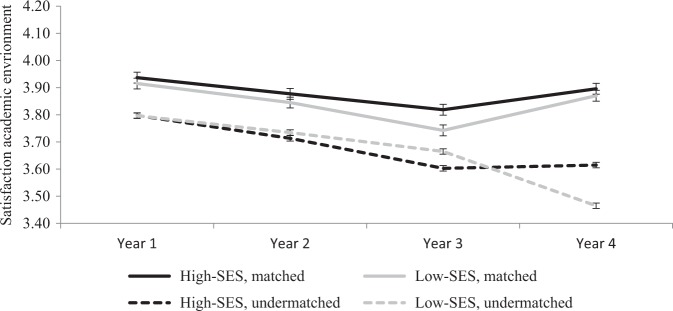
Satisfaction with the academic environment (scale 1–5) during the years in higher education among matched- and undermatched low-and high-SES students (objective SES, cross-sectional data) after propensity score matching, *N* = 8,333

In order to test whether the relation between undermatching and satisfaction develops differently among low- and high-SES students during higher education, the interaction SES × undermatching × year (in addition to all relevant two-way interactions) was added to the models presented in Table 2. Results indicate a three-way interaction between SES, undermatching, and year 4 (with year 1 as reference category) (see Table 5) with regard to satisfaction with the academic environment, but not regarding satisfaction with the social environment. In order to interpret this significant interaction, exploratory analyses for each separate year in higher education (year 1, 2, 3, and 4) were conducted, assessing simple mean comparisons. These analyses, shown in Fig. 1, reveal that this significant interaction indicates that among matched students, low- and high-SES students are equally satisfied by the fourth year of college. On the contrary, among undermatched students, the low-SES students become less satisfied than their high-SES fellow students by the fourth year of college.

**Table 5:** Estimated coefficients (Beta) predicting student satisfaction after propensity score matching: Interaction between SES, Undermatch, and Year

	Satisfaction with the social environment		Satisfaction with the academic environment	
	β	SE	β	SE
Predictors				
Undermatching (1 = undermatched)	−0.06*	0.03	−0.12**	0.04
Low-SES, objective (1 = low-SES)	−0.01	0.03	−0.04	0.0
Year (year 1 = ref)				
Year 2	−0.01	0.02	−0.05*	0.02
Year 3	−0.03	0.03	−0.08**	0.02
Year 4	−0.01	0.02	−0.05*	0.02
Three-way interactions				
Interaction SES × Undermatching × Year 2	0.00	0.03	0.00	0.02
Interaction SES × Undermatching × Year 3	0.04	0.02	0.01	0.02
Interaction SES × Undermatching × Year 4	−0.03	0.02	−0.05*	0.02
Constant	4.00**	0.03	3.85**	0.03
R-sq	0.04		0.04	
*N*	8,333		8,333	

Standardized coefficients. Both models also included college major as covariates and all two-way interactions regarding undermatching, SES, and Year (results not shown)

*p < 0.05, **p < 0.01

**Fig. 2:**
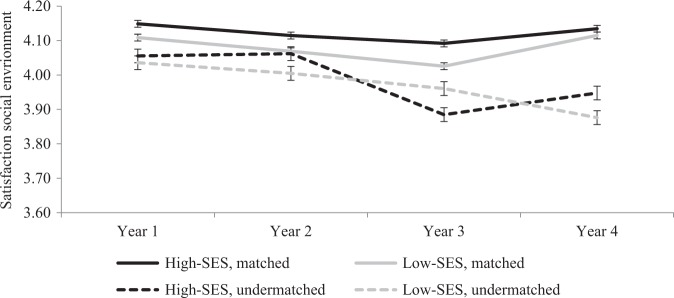
Satisfaction with the social environment (scale 1–5) during the years in higher education among matched- and undermatched low-and high-SES students (objective SES, cross-sectional data) after propensity score matching, *N* = 8,333

Importantly, these simple mean comparisons reveal that undermatched students (both low- and high SES students) are less satisfied with the social and academic environment than matched students in most academic years. In addition, these differences become more pronounced toward the fourth year in college, especially for low-SES students. In the fourth year in college, undermatched low-SES students are on average 0.43 point less satisfied with the academic environment, and 0.25 point less satisfied with the social environment than matched students on a 5-point scale (results shown in Figs. 1 and 2).

Results from all models show the same pattern when applying the alternative definition of SES (subjective SES); therefore, these results are not shown.

### Sensitivity analyses

The results of analyses after propensity score matching are presented, a technique that addresses issues concerning endogeneity. Although this procedure addresses possible confounding effects from covariates, it may at the same time harm the generalizability of the findings. After all, the matching procedure excluded participants in the control group (matched students) who have no counterpart in the treatment group because of their estimated propensity score. Therefore, a sensitivity analysis was run, by applying a conventional linear regression model predicting student satisfaction, with all covariates as controls, including the whole sample (*N* = 21,452). Results show the same pattern as the results found after applying the PSM procedure, indicating that the results are not biased by the selection based on the PSM. Therefore, results showed no evidence to suggest that the findings do not generalize to the whole student sample (see Appendix A, Table 6).

## Discussion

Undermatching, when students attend post-secondary institutions which are less selective than their academic credentials would permit, is generally considered as an undesirable outcome because of the long-term consequences for students’ job opportunities and wages (Ovink et al. 2018), especially for low-SES students, who are more likely to undermatch (Bastedo and Jaquette 2011). However, there is a gap in the literature regarding the relation between undermatching and students’ subjective experiences during college, and its relation with SES. Studying adolescents’ subjective experiences during their years in college is important because of its consequences for college completion (Bowen et al. 2009), and because of the accumulating effects that both positive and negative experiences can have in their development toward adulthood (Yoshioka and Noguchi 2009). The literature suggests that when students are undermatched, the academic demands from their institutions are misaligned with their potential (e.g., Hoxby and Turner 2013), which may lead to less satisfaction with the academic environment. However, experiences of social mismatch and feeling ‘out of place’ when low-SES students enter the most selective institutions are well documented (Jury et al. 2017). The cultural codes in less selective institutions may match better with low-SES students than the cultural codes in highly selective institutions (Deutschlander 2017). Consequently, there may be a positive relation between undermatching and satisfaction with the social environment, but only among low-SES students.

In the present study, the relation between undermatching and satisfaction, and the moderating role of SES, was investigated with a large, representative Dutch dataset that includes information about student self-reported satisfaction and student characteristics such as age, motivation, and grades during high school (*N* = 21,452 respondents). Up till now, undermatching has been studied mainly in the U.S., where undermatching has to be estimated from institutions’ selectivity levels, and students’ eligibility to these institutions. Because there are many different ways to define these constructs, concerns have raised regarding comparability and accuracy of these estimations (Rodriguez 2015). In the Netherlands, both institutional selectivity and students’ qualifications are much easier to determine, leading to highly accurate and undebatable estimations of undermatching,

The study examined satisfaction among low-SES students all of whom are eligible for the most selective institutions, but who are either in the most selective institutions (match), or in less selective institutions (undermatch). To test whether any effects are specific to low-SES students, their satisfaction was compared with the satisfaction of high-SES students in both selective and non-selective institutions. In addition, it was examined whether these relations (both for low-and high-SES students) change throughout the four years in higher education. Because pre-existing differences may confound the relation between undermatching and satisfaction, propensity score matching (PSM) was applied to test the consequence of undermatching, excluding as much as possible the confounding influences of covariates.

The present study findings show that undermatching is related to less satisfaction with the academic and social environment, and that this relation becomes stronger toward the fourth year in higher education. The study did not provide any evidence showing that undermatching is related to more satisfaction among low-SES students. These results do not only suggest that there are no benefits for low-SES students related to undermatching, undermatching even seems to have costs in terms of less satisfaction with the social and academic environment during college, especially toward the later years in higher education.

### Undermatching and academic and social satisfaction

The finding that undermatched student are less satisfied with the academic environment (i.e., the content and rigorousness of the educational program) is in line with literature that suggests that de demands from less selective institutions are misaligned with the capacities of undermatched students (Belasco and Trivette 2015). In less selective institutions, undermatched students are probably not maximizing their full potential (Hoxby and Turner 2013). Previous research has shown that students have higher chances of graduating if the quality level of their institution matches their observed skill levels (Light and Strayer 2000). The lower satisfaction after undermatching shown in the present study may be an important factor in the relation between undermatching and degree attainment.

The finding that low-SES students’ satisfaction with the social environment does not benefit from undermatching, indicates that there may also be a social mismatch when low-SES students attend less selective institutions in higher education. In the less selective institutions, the proportion low-SES students is higher than in the most selective institutions (Bastedo and Jaquette 2011) (i.e., 40% low-SES students in less selective tracks versus 25% low-SES students in the most selective tracks in the Netherlands; Dutch Inspectorate of Education 2018). As a consequence, cultural codes in less selective institutions may match better with low-SES students’ backgrounds (Walpole 2003). However, the results suggest that the larger proportion of other low-SES students seem not to elevate their satisfaction regarding experiences with the social environment. This finding may suggest a social mismatch in all higher education institutions, regardless of the level of selectivity. In addition, the finding that students who are undermatched, experience less satisfaction with the social environment, suggests that undermatching does not take along benefits in terms of satisfaction with social aspects of college, both for low- and high-SES students.

The finding that the negative relationship between undermatching and satisfaction (social and academic) seems to manifest in the later years in college, suggests that undermatching has especially consequences after students have integrated in their new college. When this negative relationship would have been strongest in their first year, this might have been related to a process of adjustment related to separation from the old situation and transition into the new college. However, the enhanced negative relationship after the phase of transition, seems to reflect how students feel about their situation once they adjusted. Although speculative, this finding may also predict a negative relationship between undermatching and satisfaction on the job market, after college.

The present study findings show that low-SES students seem not to benefit from undermatching in terms of satisfaction. Endogeneity could lead to an overestimation of the relation between undermatching and satisfaction. For example, students who are not motivated to enter higher education may be more likely to undermatch and to become dissatisfied. In addition, students who are less cognitively talented may be more likely to undermatch and become less satisfied during higher education. Nevertheless, because students with the same eligibility for the most selective institution were selected, and because PSM was applied to exclude the confounding effects of covariates, such as motivation for college and grades during high school, endogeneity is unlikely to explain the current findings.

These findings add to the body of research on the consequences of undermatching. Although there are several differences between the U.S. context and the European context in higher education, the basic principles underlying undermatching (i.e., students attend less selective institutions than their academic credentials would permit) are the same in many regards. First, both in the U.S. and in Europe, the eligibility for the most selective institutions depends on academic performance during middle adolescence. Second, an important similarity is that attending less selective institutions is on average related to less prestigious jobs and lower wages on the long term. Third, both in the U.S. and Europe, low-SES students tend to undermatch more than high-SES students. Fourth, both in the U.S. and in Europe, students’ years in college are usually spent during late adolescence, covering the same developmental stage toward early adulthood. Therefore, it is plausible that the results of the present study are generalizable to the U.S. context.

### Implications

The present study extends the knowledge about the effects of undermatching by showing that also in the short term, during college, undermatching affects students’ well-being. These results are of important because of low-SES students’ higher likelihood to undermatch (Belasco and Trivette 2015). The less positive college experiences related to undermatching may reinforce educational disadvantage for students from low-SES backgrounds. First, the lower satisfaction may have negative consequences for their college completion (Ovink et al. 2018). Second, these enduring experiences of lower satisfaction during college increase the likelihood of encountering stressful experiences related to a low socioeconomic background (Wickrama et al. 2015). This accumulation of stressful experiences during adolescence can have detrimental consequences for health and well-being in adulthood (Wickrama et al. 2016), especially for social mobile adolescents (Miller et al. 2015; Wickrama et al. 2016). In addition, the finding that the negative relationship between undermatching and satisfaction enhances toward the later years in college, suggests that this relationship manifests after students’ integration in college. Although speculative, this finding may also suggest a negative relation between undermatching and job-satisfaction after graduation.

Clearly, these findings have also implications for the formulation of policies and programs for promoting social mobility. Undermatching arises during the transition from high school to the most selective institutions and is related to a wide range of barriers (Page and Scott-Clayton 2016). Traditionally, the *knowledge deficit approach* states that students’ choice to undermatch is a result of a lack of information about application processes and college costs. Research on college choice processes indeed shows that low-SES students’ tendency to undermatch is highly related to having less access to information about institutions compared to high-SES students; low-SES students are less likely to undermatch when they receive high-quality information about their possibilities (Hoxby and Avery 2013). However, even with access to ‘perfect information’, undermatching still occurs among low-SES students (Black et al. 2015). The *preference approach* to undermatch explains this tendency by differences between low- and high-SES students in factors that students take into account during their college decision-making, like geographic factors, college fit, and opinions of relatives and peers (Black et al. 2015; Tiboris 2014). From this perspective, it has been argued that undermatching can be the result of a well-informed, autonomous decision (Tiboris 2014). In sum, both the knowledge deficit approach and the preference approach suggest that it is important to offer low-SES students high-quality information during the transition to higher education. Policy on social equality has encouraged high schools to improve information during the college choice process. The present study indicates that low-SES students should also be informed about their higher risk on lower satisfaction during the later years in higher education when they are undermatched.

### Limitations

Despite the importance of these findings, the present study has several limitations. One limitation is that the data are cross-sectional, and therefore, it cannot with certainty be concluded whether the differences between the years are actually reflecting student development during these years. For example, students who are very dissatisfied may leave higher education, resulting in a biased estimation of satisfaction from year 1 to year 4. However, student drop-out peaks after the first year in higher education: 33% switches or drops out after the first year. Yet, among students who continue after their first year, 86% obtains their diploma (Dutch Inspectorate of Education 2018). Therefore, it is plausible that the data capture student development over years, especially in the later years of higher education when drop-out rates are low. However, longitudinal data are necessary to better map this development.

Moreover, although applying PSM in order to exclude confounding effects of covariates is a highly recommended method to approach the relation between undermatching and satisfaction as close as possible, there might be unobserved confounders. For example, personality traits may also partly determine whether students undermatch or match, and these were not measured. Therefore, despite the use of PSM methodology, it is important to remain cautious with causal interpretations.

Next, certain aspects of students’ experiences in college that may influence their satisfaction, such as the possibility to engage in collaborative learning activities or in extracurricular events, were not measured. Some recent studies with small samples of first students suggest positive effects of undermatching on college experiences among first-year ethnic minorities (Fosnacht 2014, 2015; Lowry 2017), because undermatched students engage more in active and collaborative learning activities in less selective institutions. Especially black students were found to be less affected or even to benefit from undermatching. Because of limitations in the dataset, it was not possible to study the role of these college experiences, nor ethnicity, conclusively. Therefore, it is possible that undermatching can contribute positively to students’ subjective experiences when institutions offer certain social activities.

Furthermore, the reason for students to undermatch may vary across students and affect satisfaction. For example, low-SES students are likely to undermatch for the reason that they can stay closer to their family and friends (Belasco and Trivette 2015). The motives for students to undermatch may moderate the negative relation between undermatching and satisfaction. In this study, the role of the reason to undermatch could not be tested because this was not measured in the dataset. Initial differences between matched and undermatched students, such as motives to undermatch or self-efficacy, although not of explicit interest in this study, are of potential interest in future research.

## Conclusion

Students who attend less selective institutions in higher education than they are eligible for (undermatching) tend to achieve less job opportunities and lower wages in the long term than students who do not undermatch (Ovink et al. 2018). Therefore, in the literature, undermatching is mainly regarded as an undesirable outcome (e.g., Hoxby and Turner 2013), especially among low-SES students, who are more likely to undermatch (Belasco and Trivette 2015). Because of the importance of subjective experiences during adolescents for their development toward adulthood (Yoshioka and Noguchi 2009), and the gap in the literature regarding undermatching, SES, and student satisfaction, the current study tested with a large-scale cohort study 21,452 respondents (67% female) among adolescents how undermatching is related to students’ satisfaction in college by using propensity score matching (PSM, Thoemmes and Kim (2011)). Results showed a negative relation between undermatch and satisfaction with the social and the academic environment that increases toward the fourth year in college. This relation appeared to be negative for both low- and high-SES students. For example, undermatched students, regardless of their SES, are at the end of the first academic year less satisfied with the academic environment than matched students. In addition, their satisfaction lowers during the first year whereas the satisfaction among matched students remains stable. These findings have important implications for the understanding about undermatching in relation to students’ development, and for policy interventions. The higher likelihood to undermatch among students from low-SES backgrounds may reinforce their educational disadvantage, because the lower satisfaction tied to undermatching may have negative consequences for their college completion (Bowen et al. 2009), and because enduring stressful experiences during adolescence can have harmful consequences for their health and well-being in adulthood (Wickrama et al. 2016; Miller et al. 2015). The finding that the negative relationship between undermatching and satisfaction seems to increase after integration into college, suggests that the mismatch may also continue after graduation, on the labor market. Research on reasons to undermatch has shown that high-quality information is important in helping students to make appropriate decisions during their transition into higher education (Black et al. 2015; Hoxby and Avery 2013). Therefore, it is important that students, especially when they have a low-SES background, are informed about the negative relation between undermatching and satisfaction toward the later years in higher education.

## Appendix A

Table 6

**Table 6:** Sensitivity analyses: Estimated coefficients (Beta) predicting student satisfaction without propensity score matching

	Outcome: Satisfaction with the social environment		Outcome: Satisfaction with the academic environment	
	β	SE	β	SE
Predictors				
Undermatching (1 = undermatched)	−0.06**	0.01	−0.10**	0.01
Low-SES (1 = low)	−0.02*	0.01	−0.02*	0.01
Interaction: Low-SES × Undermatching	0.01	0.01	0.00	0.01
Covariates				
Gender (1 = male)	0.02*	0.01	0.01	0.01
Age	−0.04**	0.01	−0.03**	0.01
Immigrant status (1 = immigrant status)	−0.05**	0.01	−0.01	0.01
Home language (1 = Dutch)	0.04**	0.01	0.02*	0.01
Disability or chronicle disease (1 = no)	0.03**	0.01	0.02*	0.01
Average grade high school	0.05**	0.01	0.02*	0.01
Motivation before higher education	0.16**	0.01	0.17**	0.01
Constant	3.54**	0.37	3.42**	0.39
R-sq	0.07		0.06	
* N*	21452		21452	

Standardized coefficients. Both models control for college major (8 dummies, results not shown)

*SES* socioeconomic status

**p* < 0.05; ***p* < 0.01

## Footnotes

1 Although the vast majority of students in the most selective institutions in higher education are admitted after attainment of a diploma in the highest level of secondary education, a bachelors’ degree attained in a less selective institution in higher education can also give eligibility to the most selective institutions in higher education (applies to less than 4% off all students in the most selective institutions) (van den Broek et al., 2018).

2 Apart from some exceptions regarding specific majors, such as dentistry and medicine, selective institutions hold no additional requirements.
